# High Serum Level of *β*2-Microglobulin in Late Posttransplant Period Predicts Subsequent Decline in Kidney Allograft Function: A Preliminary Study

**DOI:** 10.1155/2015/562580

**Published:** 2015-11-08

**Authors:** Andriy V. Trailin, Marina V. Pleten, Tatiana I. Ostapenko, Nadiia F. Iefimenko, Olexander S. Nikonenko

**Affiliations:** ^1^Department of Laboratory Diagnostics and General Pathology, State Institution “Zaporizhzhia Medical Academy of Postgraduate Education Ministry of Health of Ukraine”, 20 Winter Boulevard, Zaporizhzhia 69096, Ukraine; ^2^Department of Transplantology, Endocrine Surgery and Cardiovascular Surgery, State Institution “Zaporizhzhia Medical Academy of Postgraduate Education Ministry of Health of Ukraine”, Zaporizhzhia Regional Hospital, 10 Orikhiv Highway, Zaporizhzhia 69050, Ukraine

## Abstract

*Background.* Identification of patients at risk for kidney allograft (KAG) failure beyond the first posttransplant year is an unmet need. We aimed to determine whether serum beta-2-microglobulin (*β*2MG) in the late posttransplant period could predict a decline in KAG function. *Methods.* We assessed a value of single measurement of serum *β*2MG at one to seventeen years after transplantation in predicting the estimated glomerular filtration rate (eGFR) and the decline in eGFR over a period of two years in 79 recipients of KAG. *Results.* At baseline serum *β*2MG concentration was higher (*P* = 0.011) in patients with allograft dysfunction: 8.67 ± 2.48 *µ*g/mL versus those with satisfactory graft function: 6.67 ± 2.13 *µ*g/mL. Higher *β*2MG independently predicted the lower eGFR, the drop in eGFR by ≥25% after one and two years, and the value of negative eGFR slope. When combined with proteinuria and acute rejection, serum *β*2MG had excellent power in predicting certain drop in eGFR after one year (AUC = 0.910). In conjunction with posttransplant time serum *β*2MG had good accuracy in predicting certain eGFR drop after two years (AUC = 0.821). *Conclusions.* Elevated serum *β*2MG in the late posttransplant period is useful in identifying patients at risk for rapid loss of graft function.

## 1. Introduction

Kidney transplantation is the most effective method of treatment for patients with terminal kidney insufficiency. At present the rate of one-year kidney allograft survival exceeds 95% [1] and the preservation of allograft function in the late period after transplantation has become the main challenge [1, 2]. Identification of patients at risk for kidney graft failure beyond the first posttransplant year is a prerequisite for developing strategies for saving graft function and improving its survival [3–6]. Many recent studies have focused on higher serum creatinine and lower glomerular filtration rate (GFR) within the first posttransplant year as surrogate predictors of inferior graft survival [3, 7–10]. The rate of decline in GFR also predicts graft failure [3, 9] and can be even more useful surrogate endpoint [11, 12]. However, the ability of allograft function in the first year after transplantation to predict the long-term results is questionable [3, 13]. Furthermore, serum creatinine and estimated GFR (eGFR) are now considered by some authors as not quite sensitive and specific markers of kidney function [14–16]. Additional surrogate predictors of decline in allograft function in the late posttransplant period can help better identify patients at risk for failure in order to improve their monitoring and allow early intervention.

*β*2-microglobulin (*β*2MG) is a membrane protein associated with class I major histocompatibility complex proteins and is, therefore, found on the surface of all nucleated cells. Under physiological conditions, *β*2MG is produced at a constant rate and is eliminated through the kidney. The low molecular weight (11.800 Da) allows *β*2MG to pass through the glomerular membrane, but it is almost completely reabsorbed in the proximal tubules [17]. Several authors report that measurement of the serum *β*2MG concentration in native kidney diseases estimates GFR as serum creatinine does [16, 18, 19] and even supersedes it [15, 20, 21]. Serum *β*2MG level increases because of intensification of its extrarenal synthesis in conditions such as systemic inflammation, some acute viral infections, and a number of malignancies [17, 19, 22–25]. Data about *β*2MG as a marker of kidney graft function are limited and contradictory [26–28]. Astor et al. have recently demonstrated that high serum *β*2MG at discharge predicted kidney graft loss [29], but detailed retrospective information might not be available for all patients. The association between serum *β*2MG and the rate of decline in kidney allograft function remains obscure. The aim of the present study was to determine whether serum beta-2-microglobulin at different intervals of late posttransplant period could predict the decline in GFR over a follow-up period of two years.

## 2. Materials and Methods

### 2.1. Study Population

During the four-month period (from September till December, 2012), in total, 90 Caucasian patients receiving a kidney allograft in Zaporizhzhia transplantation center between January 1995 and September 2011 and willing to participate were recruited. A total of 79 patients, 47 males and 32 females, aged 16 to 59, who fulfilled the inclusion criteria, were enrolled in the study. The criteria of inclusion to the study were the following: the adult kidney allograft recipient, male or female, with primary transplantation from related or deceased donor, with allograft survival of at least one year and eGFR not less than 15 mL/min/1.73 m^2^. The criteria of exclusion were regular dialysis, acute kidney injury, diseases of immune system, solid tumours, and clinical signs of acute infections. All recipients received triple maintenance immunosuppressive therapy consisting of calcineurin-inhibitor (cyclosporine or tacrolimus), antiproliferative agent (mycophenolate mofetil or azathioprine), and steroid. All participants gave their informed written consent. This research was approved by local ethics committee and carried out in accordance with the ethical standards laid down in the Declaration of Helsinki (2013 version) and the Declaration of Istanbul.

### 2.2. Laboratory Methods

Serum was obtained from the venous blood taken in the morning from patients in a fasting condition. For measuring of *β*2MG serum had been frozen and stored at minus 40°С. Simultaneously, freshly voided morning urine samples were collected and centrifuged at 3000 rpm for 15 min. Urine specific gravity was measured with a urinometer. We measured serum concentration of urea by the urease method, urine protein level by the pyrogallol red-molybdate method, and serum and urine creatinine concentration by the Jaffe method. All kits were supplied by “Filisit-Diagnostics,” Ukraine. The absorbance was measured on spectrophotometer “APEL” (Japan). The results of total protein in urine spot were normalized to urinary creatinine. As a threshold of normalized proteinuria we considered the level of 15 mg/mmol [12]. For GFR estimation we used a four-variable equation derived from the Modification of Diet in Renal Disease (MDRD) Study [30]. Quantitative determination of *β*2MG in serum samples was performed by enzyme-linked immunosorbent assay (kit supplied by Orgentec GmbH, Germany) according to the manufacturer's protocol. The absorbance was measured using a plate reader “Tecan” Sunrise (Austria). Mean reference value given for serum *β*2MG is 0–3.0 *μ*g/mL.

### 2.3. Risk Factors and Outcomes Examined

In this study we assessed a prospective value of a cross sectional measurement of serum *β*2MG. Archival patient records and outpatient cards were used to obtain more information on major risk factors and evolution of allograft function. Data were collected on recipient's age, gender, and presence of chronic arterial hypertension, defined as a regular intake of antihypertensive drugs. We also obtained the information related to transplantation: donor source, type of immunosuppression, initial graft function, acute rejection episodes, and time after transplantation. At enrollment the arterial pressure was measured in a sitting position after a 10-minute rest period. For linear regression analysis the initial allograft function was classified as follows: immediate function (0 points), slow graft function, that is, serum creatinine reduction from transplantation to day seven <70% (1 point), delayed graft function (DGF), that is, need for at least one time dialysis in the first seven days after surgery (2 points), and DGF that required more than one dialysis procedure (3 points). Acute rejection was defined by the need for treatment, with or without biopsy confirmation. For linear regression analysis episodes of acute rejection were classified as follows: absence of acute rejection (0 points), early (<3 months) acute rejection successfully treated by steroid therapy (1 point), late acute rejection successfully treated by steroid therapy (2 points), acute rejection with need for antilymphocyte antibody therapy (3 points), and resistant to therapy acute rejection with noncomplete recovery (4 points). Patients, enrolled in this study, were followed for two years until death/return to dialysis or until December 2014. In the course of follow-up period GFR was estimated annually. During the 2nd year of follow-up, a total of three deaths with functioning graft occurred and five grafts failed. For five patients, who returned to dialysis, we imputed a GFR of 10 mL/min/1.73 m^2^. The annualized change (slope) in eGFR (mL/min/1.73 m^2^/year) over a period of two years was calculated for each patient, having three eGFR values, by the linear mixed effects model with varying intercept and slope. We determined the proportion of patients having an eGFR slope ≥ −1 mL/min/1.73 m^2^/year and the proportion of patients having an eGFR drop of ≥25% from baseline, since both measures indicate progressive loss of kidney function [12]. We also calculated the frequency of patients who showed improvement in GFR (increase in eGFR ≥ 1 mL/min/1.73 m^2^/year and increase in eGFR ≥25% from baseline). The endpoints of the study were the eGFR and the drop in eGFR of ≥25% from baseline after one and two years of follow-up, and the slope of eGFR.

### 2.4. Statistics

Normally distributed data are expressed as mean ± SD; the results were compared with Student's *t*-test; Pearson's coefficient of correlation (*r*) was determined where appropriate. Continuous nonparametric data are expressed as the median (interquartile range); for comparison we used Mann-Whitney's *U*-test; Spearman's correlation coefficient (*R*) was calculated where appropriate. Frequency data are expressed as percentages and for comparison we applied the Chi-square test. To identify predictors of eGFR after one and two years and those of eGFR slope, multiple linear regression was used. Normalized proteinuria and mean arterial pressure had non-Gaussian distribution and were natural log-transformed. The predictors that significantly correlated with dependent variable were tested for multicollinearity and excluded when appropriate. Predictive variables, if significantly associated (*P* < 0.05) with the dependent variable in simple linear regression analysis, were included in multivariate model with forward stepwise selection. To identify independent predictors of a certain drop in eGFR (≥25% from baseline [12]) after one and two years of follow-up we used univariate logistic regression analysis. Then multivariate analysis was performed with only those predictive variables that demonstrated individual *P* values <0.05. In addition, we calculated areas under the receiver operating characteristic curves (AUC) to assess the capability of serum *β*2MG and other variables of interest to discriminate patients with a drop in eGFR ≥25% after one and two years of follow-up. We used logistic regression models to estimate combinations of serum *β*2MG and other variables of interest and evaluated the discriminatory ability of the combinations with the AUC. AUCs were compared using the DeLong test. Statistica 7.0 (StatSoft Inc., Tulsa, USA), SPSS (version 19.0 SPSS Inc., Chicago, USA) and Medcalc V.14.8.1 (MedCalc Software bvba, Ostend, Belgium) packages were used for statistical analyses. Statistical significance was set at *P* < 0.05.

## 3. Results

### 3.1. Baseline Characteristics of Patients

The mean eGFR at baseline was 50.4 ± 19.7 mL/min/1.73 m^2^. Most patients were in the third (*n* = 49) or in the second (*n* = 21) stages of chronic kidney disease (CKD). We decided to categorize patients according to the median eGFR into “Satisfactory Function” and “Dysfunction” subsets with eGFR >44 mL/min/1.73 m^2^ and eGFR ≤44 mL/min/1.73 m^2^, respectively. We gained confidence in the correctness of the chosen approach since the eGFR of 44 mL/min/1.73 m^2^ is a threshold between CKD stages G3a and G3b. The demographics and clinical characteristics of patients are depicted in Table 1. The majority of patients received a kidney from a deceased donor and they were on CsA-based immunosuppression. No differences in gender, age, or type of immunosuppression were observed between subsets of recipients. Most of the patients (62.5% and 74.4% of the Dysfunction group and Satisfactory Function group, resp.) were transplanted from one to ten years ago. Time after transplantation exceeded 10 years in 35% of patients from the Dysfunction group and in 15.4% of patients with satisfactory function (*P* = 0.045). Accordingly, the time after transplantation was significantly longer in the Dysfunction group (Table 1). In the Dysfunction group impairment of initial function and acute rejection episodes were significantly more common. Only one patient had late acute rejection (at 3.5 years), whereas the remaining patients had early acute rejection episodes (within 3 months). The mean arterial pressure and the percentage of patients regularly receiving antihypertensive therapy were significantly higher in the Dysfunction group. The differences between the main laboratory parameters of graft status (serum creatinine, eGFR, and normalized proteinuria) were highly significant (Table 1).

### 3.2. *β*2MG Level and Its Correlation with Allograft Function at Baseline

Serum *β*2MG level remained in the reference ranges (0–3.0 *μ*g/mL) only in one patient, whereas it was elevated in the other patients. *β*2MG concentration was significantly higher (*P* = 0.011) in the subset of patients with allograft dysfunction: 8.67 ± 2.48 *μ*g/mL versus 6.67 ± 2.13 *μ*g/mL in Satisfactory Function subset. The clinical variables: type of the donor, age and gender of the recipient, characteristics of acute rejection episodes, type of initial graft function, mean arterial pressure, and time after transplantation did not correlate with the serum levels of *β*2MG (*P* > 0.05). Only serum creatinine concentration (*r* = 0.466, *P* = 0.002), eGFR (*r* = −0.338, *P* = 0.033), and normalized proteinuria (*r* = 0.388, *P* = 0.013) significantly correlated with serum *β*2MG levels.

### 3.3. The Evolution of Allograft Function and the Predictive Variables

**Figure 1:**
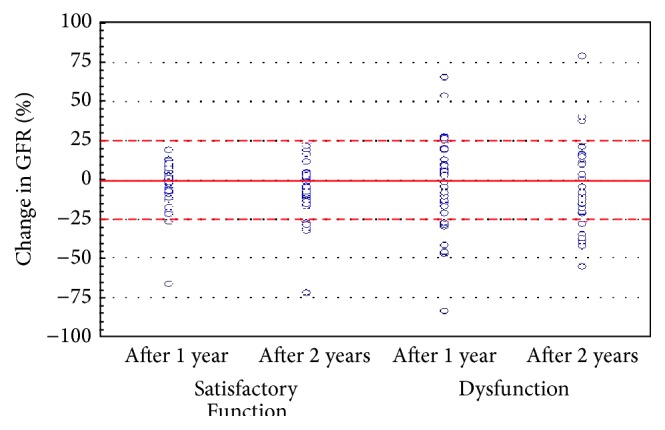
Scatterplot of patients from Satisfactory Function and Dysfunction subsets according to the change in glomerular filtration rate (GFR).

After one year of follow-up, the mean eGFR did not significantly change in the total group of patients: 49.2 ± 19.7 mL/min/1.73 m^2^ compared to eGFR at baseline (*P* = 0.289). Twenty percent of the patients from the Dysfunction group and only five percent of patients from the Satisfactory Function group (*P* = 0.047) demonstrated decline in eGFR by ≥25% from baseline after 1 year (Figure 1). Eight patients had chronic allograft dysfunction, but only in two cases the diagnoses were confirmed histologically. One patient had chronic active T-cell-mediated rejection and the second patient had chronic nephrotoxicity in combination with hypertensive nephropathy. We also observed progression of heart failure from New York Heart Association functional class II to class III in two other patients, which might cause worsening of graft function. Fifteen percent of patients from the Dysfunction group showed the increase in eGFR by ≥25% (Figure 1) after 1 year, whereas such patients were absent in the Satisfactory Function group (*P* < 0.001). The presumed causes of improving graft function were conversion of immunosuppression (from cyclosporine to tacrolimus in two patients and from mycophenolate mofetil to everolimus in two other patients), effective antibiotic treatment of urinary tract infection (1 patient), and increase of renal perfusion after coronary artery bypass (1 patient). During the 2nd year of follow-up, a total of three deaths with functioning graft occurred and a total of five grafts failed. The cause of allograft failure was verified histologically in selected patients and we found chronic active antibody-mediated rejection (1 patient), chronic pyelonephritis (1 patient), and* de novo* glomerulonephritis (1 patient). After two years of follow-up the mean eGFR in the total group of patients significantly decreased (46.3 ± 20.6 mL/min/1.73 m^2^) compared with that at baseline (*P* = 0.002). This decline was also significant compared to eGFR after one year (*P* < 0.001). 22.5% of patients from the Dysfunction group and 13% of patients from the Satisfactory Function group displayed the decrease in eGFR by ≥25% compared to baseline (*P* = 0.260) (Figure 1). We did not observe any new intercurrent events during this period. Only 10% of patients (all from the Dysfunction group) showed the increase in eGFR by ≥25% compared with that at baseline (*P* = 0.043) (Figure 1). The eGFR slope in the overall group of patients was −2.0 ± 5.7 mL/min/1.73 m^2^/year (median: −2.0 mL/min/1.73 m^2^/year). In Satisfactory Function subset the slope was −2.4 ± 6.3 mL/min/1.73 m^2^/year (median: −1.5 mL/min/1.73 m^2^/year), which did not significantly differ from Dysfunction subset (*P* = 0.519): −1.6 ± 5.2 mL/min/1.73 m^2^/year (median: −2.0 mL/min/1.73 m^2^/year).

The eGFR decreased by ≥1 mL/min/1.73 m^2^/year in 60% of patients from the Dysfunction group and in 62% of patients with satisfactory graft function (*P* > 0.05). At the same time, the eGFR increased by ≥1 mL/min/1.73 m^2^/year in 30% of patients from the Dysfunction group and in 31% of patients with satisfactory function (*P* > 0.05). In the course of the study the relationship between the eGFR and serum *β*2MG was increasing: *r* = −0.338 (*P* = 0.033) at baseline to *r* = −0.538 (*P* < 0.001) at one year and *r* = −0.527 (*P* < 0.001) at two years after enrollment.

Table 2 summarizes the linear regression results for predictors of eGFR at different time points. The clinical variables, mean arterial pressure, normalized proteinuria, impaired initial graft function, and acute rejection, were associated with lower eGFR at baseline in univariate analysis. In multivariate analysis, only higher mean arterial pressure and higher normalized proteinuria were independent predictors of lower eGFR (Table 2). To determine the independent effect of *β*2MG on the evolution of kidney allograft function we also included clinical variables in the regression equation. The following variables significantly influenced the eGFR after one year in univariate analysis: eGFR at baseline, serum *β*2MG concentration, normalized proteinuria, mean arterial pressure, time after transplantation, and initial graft function (Table 2). The same variables and, in addition, acute rejection significantly influenced the eGFR after two years in univariate analysis (Table 2). In multivariate model, only lower eGFR at baseline and higher serum *β*2MG concentration were independent predictors of lower eGFR after one and two years of follow-up. The values of the adjusted coefficient of determination *R* ^2^ = 0.712 and Fisher statistics (*F* = 48.0, *P* < 0.001) indicated the good quality of models for prediction of the eGFR after one year as well as after two years: *R* ^2^ = 0.665, *F* = 26.2, and *P* < 0.001.

Only higher serum concentration of *β*2MG was a predictor of more negative slope of eGFR in linear regression analysis (*β* = −0.222, SE = 0.111, and *P* = 0.049). None of other variables, including the eGFR at baseline, correlated with the magnitude of eGFR slope.

Table 3 provides details of logistic regression analysis for the value of *β*2MG level and other laboratory and clinical parameters to predict the eGFR decline by ≥25% after one and two years of follow-up. Higher normalized proteinuria and serum *β*2MG as well as previous acute rejection episodes were individually predictive for decline in eGFR of ≥25% after one year of follow-up (Table 3), wherein only serum *β*2MG retained its predictive value in multivariate model. With respect to the graft function after two years of follow-up both higher serum *β*2MG and longer time after transplantation were associated with higher odds ratio of certain eGFR drop (Table 3).

The discriminating ability of each variable, considered significant by simple logistic regression, for the drop in eGFR ≥25% after one and two years of follow-up was determined by the AUC analysis. As shown in Table 4, only serum *β*2MG was the significant predictor for the decline in eGFR by ≥25% after one year. The combination of normalized proteinuria, acute rejection, and serum *β*2MG resulted in an AUC of 0.910 in predicting certain eGFR drop after one year (Table 4), which indicated excellent discriminating ability. This AUC showed higher predictive power than AUC for normalized proteinuria (*P* = 0.0022) and acute rejection alone (*P* = 0.0003) but did not significantly differ from the AUC for serum *β*2MG (*P* = 0.070). Serum *β*2MG and time after transplantation exhibited only poor and fair discriminatory power, respectively, in predicting the eGFR decline by ≥25% after 2 years of follow-up (Table 4). The AUCs for these two variables were not significantly different from each other (*P* > 0.05). The combination of serum *β*2MG and time after transplantation yielded the AUC of 0.821, indicating good predictive power for certain eGFR drop after two years (Table 4). The AUC of this combined model was higher than the AUC for each predictor alone, but not significantly (*P* > 0.05).

## 4. Discussion

In this study we found the association between serum level of *β*2MG in the late posttransplant period and a subsequent decline in kidney allograft function within a short time-frame. Serum *β*2MG not only was associated with lower eGFR, but also predicted the value of negative slope in eGFR and the certain decline in eGFR of ≥25% from baseline. It is of importance that predicted drop in eGFR occurred in a short time-frame of two years and even one year. We also found the fair and poor accuracy of serum *β*2MG after one and two years, respectively, in discriminating patients with eGFR drop of ≥25%. However, in conjunction with normalized proteinuria at baseline and history of acute rejection, serum *β*2MG had excellent power in predicting certain drop in eGFR after one year. When combined with time after transplantation serum *β*2MG had good power in predicting the eGFR drop of ≥25% after two years.

As only higher serum creatinine, lower eGFR at baseline, and proteinuria were associated with higher serum *β*2MG concentration, we believe that the current allograft status is the main factor that influences serum *β*2MG. Several studies evaluating the role of serum *β*2MG as a marker of kidney function reported that serum *β*2MG concentration increased when renal function decreased both in transplanted patients [26, 27] and in patients with CKD [16, 17, 19, 31, 32]. Other authors stated that serum *β*2MG estimated GFR even more accurately compared to serum creatinine [15, 20, 21]. Woo et al. [33] concluded that the increase of serum *β*2MG concentration evidenced a glomerular pathology, which has recently been recognized as the main cause of chronic allograft dysfunction [6]. Meanwhile, observed weak correlation (*r* = 0.466) between serum *β*2MG and creatinine (though their sieving coefficients are close to unity [33]) suggests that another reason for the increase of serum *β*2MG concentration might be transplant complications and comorbidities leading to an increase of *β*2MG synthesis. The increase of serum *β*2MG after kidney transplantation can be a marker for cytomegalovirus infection [23], lymphoproliferative disease [24, 25], acute kidney allograft rejection [28, 34, 35], CsA-nephrotoxicity [35], and cardiovascular diseases [31]. These data imply that patients with elevated serum level of *β*2MG require closer monitoring.

Most of the patients displayed decline in the eGFR during the follow-up, whereas others exhibited increase or stabilization. After two years of follow-up the average eGFR decreased significantly compared to baseline. Our data are consistent with the conception of several patterns of graft function evolution over time [36, 37]: achieving optimal or suboptimal function with subsequent stabilization or decline. In addition, several papers reported the ability of grafts to increase GFR after six months or one year [3, 8, 38, 39]. The capacity to increase GFR in much later period after transplantation, as we have shown, suggests that even in late posttransplant period many allografts retain a substantial functional reserve and timely therapy can be effective.

Only lower eGFR at baseline and higher serum *β*2MG concentration were shown both as independent predictors of lower eGFR after one and two years of follow-up. The observed associations were independent from potential confounders. About 71% and 67% of the variances in the eGFR at one and two years after enrollment, respectively, can be explained by combined influence of the eGFR at baseline and serum *β*2MG concentration, which allows the use of this model in the future on other patients. Further, we evaluated the predictors of the slope of eGFR, which might be even more accurate and an earlier indicator of chronic allograft injury and better predict allograft function and survival [11, 12]. Patients in the whole group as well as in their subsets demonstrated the negative average slope of eGFR. A more negative slope of eGFR was typical for patients with higher concentration of serum *β*2MG, but it was independent from eGFR at baseline. The independence of the slope from the absolute level of GFR [38] and even opposite relationship between the GFR intercept and slope [39, 40] were reported earlier. At the same time, Sijpkens et al. [36] found a significant correlation between low creatinine clearance at 6 months and negative slope. But it should be mentioned that in the study by Sijpkens et al. [36] patients were transplanted between 1979 and 1983, before the introduction of CsA, when the deleterious effect of acute rejection episodes was more pronounced. Our findings along with the literature data imply that eGFR alone is not quite an accurate predictor of kidney graft failure. This is supported by the recent paper of Park et al. [3], describing that 41% of allografts with a satisfactory function at one year (eGFR ≥40 mL/min/1.73 m^2^) will progressively lose it and cease to function in the range of one to five years. In this regard *β*2MG, which accurately reflects graft function and provides information about additional injuries to the graft, might improve prediction [29].

We also demonstrated that after adjustment for confounding variables in logistic regression serum *β*2MG predicted a drop in eGFR by ≥25% after one and two years of follow-up. Thus, in multivariate model, for a one-unit increase in serum *β*2MG concentration, the odds ratio for certain drop in eGFR was 1.54 and 1.41 after one and two years, respectively. Serum *β*2MG alone showed a fair discriminatory power for certain decline in eGFR after one year. However, when combined with normalized proteinuria and acute rejection, serum *β*2MG further enhanced the quality of prognosis and displayed an excellent predictive performance. As long as the AUC for combination of variables did not significantly differ from that for *β*2MG alone, but it was higher than the AUCs for normalized proteinuria and acute rejection alone (*P* < 0.05), we conclude that serum *β*2MG provides absolutely essential data to the predictive model. Serum *β*2MG alone exhibited poor power to discriminate patients with certain decline in eGFR after two years. However, in conjunction with the time after transplantation serum *β*2MG enhanced the quality of prognosis and displayed good predictive performance. Thus, when combined with selected laboratory and clinical variables serum *β*2MG can predict a certain drop in GFR with high sensitivity and specificity. A negative impact of longer time after transplantation on the stability of graft function can be attributed to larger cumulative burden of injury with increasing time [41] and to the effect of transplant era. Thus, as reported in paper by Kasiske et al. [42], the rate of decline in kidney graft function significantly improved in recent years due to improvement in patients' care and treatment strategies.

We also identified proteinuria, higher mean arterial pressure, impaired initial function, and acute rejection episodes as clinical predictors of lower allograft function in the late posttransplant period. These variables are a combination of immunologic and nonimmunologic factors, associated with the early posttransplant events and chronic damage to the graft that fit into the conception of etiology and pathogenesis of chronic allograft dysfunction [11, 13, 37]. But their influence was significant only in univariate model, before adjustment for eGFR and serum *β*2MG. Our results imply that lower eGFR and higher serum *β*2MG in the late posttransplant period are surrogate markers of harmful effects on kidney allograft. None of clinical predictive variables (except time after transplantation) were independently associated with the rate of decline in kidney allograft function, whereas in several papers [36, 38, 40, 42] authors found the association of donor, recipient, and transplant variables with progressive loss of renal function in the course or after the 1st posttransplant year. Unlike these studies, our patients were in much later posttransplant period: from one to seventeen years. We followed recipients only for two years, but about 30% of patients exhibited positive eGFR slope. Also our results suggest that effects of early acute rejection and impaired initial function might not be so deleterious in the late posttransplant period. As highlighted by Meier-Kriesche et al. [43], two phases in evolution of allograft dysfunction exist. Technical problems and acute rejection have the greatest influence on the allograft function and survival in the early phase, whereas effects of chronic rejection, recurrent diseases, and nephrotoxicity predominate in the late phase [6]. Observed in recent years modest improvement in the allograft survival was achieved mainly due to improving of short-term outcomes, in particular, because of decrease in the incidence of acute rejection [1, 43]. Therefore, it is necessary to identify risk factors which are harmful for graft in the late posttransplant period as well as their surrogates. Also, since there are a number of variables that might not be available early after transplantation but, when available, can change the approach to therapy, we believe that information provided by late posttransplant tests, particularly, by serum *β*2MG, might be of use.

Our results suggest some more clinical and theoretical implications. Association of elevated serum *β*2MG with both low eGFR and decline in eGFR from baseline might have considerable clinical impact, since these surrogates are directly related to inferior allograft survival [3, 7, 11, 12] and, when combined, the negative effect is even more pronounced [9]. Predictably, a positive slope is associated with improved allograft survival [8]. Thus, the results lead us to believe that serum *β*2MG represents a link between low intercept and negative slope of GFR and can be an important surrogate marker for the progression of kidney allograft dysfunction. Thereby, our findings expand results of previous studies on the relationship between GFR intercept and slope and their predictive variables. It is also worthwhile to highlight that certain decline in GFR over one and two years, predicted by serum *β*2MG, indicates rapid progression of kidney allograft dysfunction [12]. To our knowledge, this is the first study to introduce late posttransplant serum *β*2MG for the prediction of decline in kidney graft function in the short time-frame. This study, however, has a number of limitations. First, it was a single-center study carried out on a small group of Caucasian patients and restricted to only two years of follow-up period. So our conclusions might have only preliminary character and should be confirmed with a larger cohort representative of the general kidney transplant population. Second, we did not measure GFR directly but relied on estimating GFR from MDRD equation, which, however, is allowable in accordance with the recommendations of recent guidelines [12]. Third, the creatinine measurements were not calibrated with respect to a reference laboratory. However, the effect of possible calibration error would equally influence the results of measurements in all patients and, therefore, cannot explain the observed differences in eGFR values. The fourth limitation is that GFR estimation was not paired with *β*2MG measurement at all time points. These data could help elucidate the processes leading to graft failure and understand whether the serum concentration of *β*2MG was mainly determined by the level of kidney function or increased protein synthesis. Finally, we can only assume the etiology of dysfunction, because allograft biopsy was performed only in individual patients. It is possible that future combination of serum *β*2MG measurement with biopsy results will improve diagnostics and prediction of kidney transplant pathology.

## 5. Conclusions

The overall results show that higher serum level of *β*2MG after a single measurement at different intervals of late posttransplant period independently predicts the lower eGFR and the drop in eGFR by ≥25% after one and two years of follow-up, as well as the value of negative annualized change in eGFR. With respect to its discriminative characteristics serum *β*2MG predicts the eGFR drop of ≥25% after one year and two years with fair and poor accuracy, respectively. However, when combined with normalized proteinuria at baseline and history of acute rejection serum *β*2MG demonstrated excellent power in predicting certain eGFR drop after one year. In conjunction with time after transplantation serum *β*2MG had good accuracy in predicting certain eGFR drop after two years. Thus, higher serum *β*2MG is a risk factor for decline in eGFR, particularly with respect to certain drop in eGFR after one year of follow-up. These observations highlight the potential importance of elevated serum *β*2MG in the late posttransplant period in identifying a group of transplant patients who are at risk for rapid loss of graft function and might benefit from early therapeutic interventions.

## Figures and Tables

**Table 1:** Baseline characteristics of the study population.

Parameter	Satisfactory Function subset	Dysfunction subset	*P* value
	*N* = 39	*N* = 40	
Recipient age at baseline, years	37 ± 12^*∗*^	40 ± 11	0.329
Recipient gender, *n* (%)			0.573
Male	21 (54)^‡^	19 (48)	
Female	18 (46)	21 (52)	
CNI, *n* (%)			0.105
CsA	34 (87)	29 (73)	
Tacrolimus	5 (13)	11 (27)	
Antiproliferative agent, *n* (%)			0.190
Azathioprine	3 (8)	7 (18)	
Mycophenolate mofetil	36 (92)	33 (72)	
Type of donor, *n* (%)			0.958
Deceased	32 (82)	33 (83)	
Living related	7 (18)	7 (17)	
Impaired initial function, *n* (%)	2 (5.1)	10 (25)	0.014
Previous acute rejection, *n* (%)	5 (12.8)	14 (35)	0.021
Time after transplantation (months)	72.6 ± 46.9	98.2 ± 57.7	0.034
Serum creatinine (*µ*mol/L)	117.7 ± 29.5	195.7 ± 49.7	<0.001
eGFR at baseline (mL/min/1.73 m^2^)	66.7 ± 13.4	34.3 ± 7.9	<0.001
Serum urea (mmol/L)	7.4 ± 2.3	11.9 ± 3.4	<0.001
Proteinuria/creatinine (mg/mmol)	3.6 (2.7–7.9)^†^	11.8 (4.5–21.8)	<0.001
Proteinuria/creatinine > 15 mg/mmol, *n* (%)	2 (5.1)	15 (37.5)	<0.001
Diuresis (mL/day)	1428 ± 243	1498 ± 372	0.331
Urine specific gravity	1012 ± 4	1007 ± 4	<0.001
Mean arterial pressure at baseline (mm Hg)	103 ± 9	113 ± 9	<0.001
Treated hypertension at baseline, *n* (%)	12 (30.8)	28 (70.0)	<0.001

CNI: calcineurin inhibitors; CsA: cyclosporine A; eGFR: estimated glomerular filtration rate; *N*: number of patients investigated; ^*∗*^mean ± standard deviation, Student's *t*-test used for comparison; ^†^median (interquartile range), Mann-Whitney's *U*-test used for comparison; ^‡^numbers (percentages), Chi-square tests used for comparison.

**Table 2:** Significant predictors of eGFR at different time points of late posttransplant period^*∗*^.

Predictive variables	Univariate linear regression			Multivariate linear regression		
	Beta	SE	*P* value	Beta	SE	*P* value
eGFR at baseline (mL/min/1.73 m^2^), *N* = 79						
Log proteinuria/creatinine^†^	−0.488	0.104	<0.001	−0.406	0.153	0.012
Impaired initial function^‡^	−0.235	0.112	0.040			
Log mean arterial pressure^†^	−0.502	0.099	<0.001	−0.332	0.153	0.037
Previous acute rejection^‡^	−0.241	0.112	0.035			

eGFR after 1 year of follow-up (mL/min/1.73 m^2^), *N* = 79						
eGFR at baseline	0.870	0.057	<0.001	0.721	0.091	<0.001
Log proteinuria/creatinine^†^	−0.476	0.105	<0.001			
Serum *β*2MG	−0.538	0.137	<0.001	−0.291	0.091	0.003
Impaired initial function^‡^	−0.233	0.113	0.042			
Log mean arterial pressure^†^	−0.436	0.103	<0.001			
Time after transplantation	−0.262	0.111	0.021			

eGFR after 2 years of follow-up (mL/min/1.73 m^2^), *N* = 71						
eGFR at baseline	0.838	0.063	<0.001	0.649	0.101	<0.001
Log proteinuria/creatinine^†^	−0.473	0.105	<0.001			
Serum *β*2MG	−0.527	0.138	<0.001	−0.302	0.098	0.004
Impaired initial function^‡^	−0.230	0.113	0.030			
Log mean arterial pressure^†^	−0.447	0.103	<0.001			
Time after transplantation	−0.332	0.108	0.003	−0.148	0.097	0.135
Previous acute rejection^‡^	−0.261	0.112	0.023			

*β*2MG: *β*2-microglobulin; eGFR: estimated glomerular filtration rate; beta: standardized regression coefficient; SE: standard error of beta; *N*: number of patients investigated; ^*∗*^only variables that significantly influenced the eGFR in univariate analysis are included; ^†^natural log-transformed variables; ^‡^impaired initial function and acute rejection scored in points.

**Table 3:** Significant predictors of eGFR decline by ≥25% after one and two years of follow-up^*∗*^.

Predictive variables	Univariate logistic regression			Multivariate logistic regression		
	OR	CI	*P* value	OR	CI	*P* value
eGFR decline by ≥25% after 1 year of follow-up, *N* = 10						
Proteinuria/creatinine	1.03	1.00–1.07	0.038	1.03	0.99–1.07	0.158
Serum *β*2MG	1.51	1.10–2.07	0.010	1.54	1.05–2.26	0.025
Previous acute rejection	4.82	1.12–20.84	0.032	2.24	0.36–13.85	0.378

eGFR decline by ≥25% after 2 years of follow-up, *N* = 12						
Serum *β*2MG	1.36	1.06–1.76	0.016	1.41	1.06–1.87	0.016
Time after transplantation	1.02	1.00–1.03	0.006	1.02	1.00–1.03	0.006

*β*2MG: *β*2-microglobulin; eGFR: estimated glomerular filtration rate; OR: odds ratio; CI: 95% confidence interval; *N*: number of patients with certain decline in eGFR; ^*∗*^only variables that significantly influenced the eGFR decline in univariate analysis are included.

**Table 4:** AUC for potential markers of eGFR decline by ≥25% after one and two years of follow-up^*∗*^.

Predictive variables	AUC	CI	*P*-level
eGFR decline by 25% after 1 year of follow-up, *N* = 10			
Proteinuria/creatinine	0.724	0.606–0.823	0.056
Previous acute rejection	0.675	0.559–0.777	0.055
Serum *β*2MG	0.724	0.612–0.819	0.026
Proteinuria/creatinine + previous acute rejection + serum *β*2MG	0.910	0.818–0.965	<0.001

eGFR decline by 25% after 2 years of follow-up, *N* = 12			
Serum *β*2MG	0.668	0.553–0.770	0.047
Time after transplantation	0.712	0.599–0.808	0.008
Serum *β*2MG + time after transplantation	0.821	0.719–0.899	<0.001

*β*2MG: *β*2-microglobulin; eGFR: estimated glomerular filtration rate; AUC: areas under the receiver operating characteristic curve; CI: 95% confidence interval; *N*: number of patients with certain decline in eGFR; ^*∗*^only variables that significantly influenced the eGFR decline in univariate logistic regression are included.
